# The Inducible Response of the Nematode *Caenorhabditis elegans* to Members of Its Natural Microbiota Across Development and Adult Life

**DOI:** 10.3389/fmicb.2019.01793

**Published:** 2019-08-07

**Authors:** Wentao Yang, Carola Petersen, Barbara Pees, Johannes Zimmermann, Silvio Waschina, Philipp Dirksen, Philip Rosenstiel, Andreas Tholey, Matthias Leippe, Katja Dierking, Christoph Kaleta, Hinrich Schulenburg

**Affiliations:** ^1^Research Group Evolutionary Ecology and Genetics, Zoological Institute, Christian-Albrechts-Universität zu Kiel, Kiel, Germany; ^2^Research Group Comparative Immunobiology, Zoological Institute, Christian-Albrechts-Universität zu Kiel, Kiel, Germany; ^3^Research Group Medical Systems Biology, Institute for Experimental Medicine, Christian-Albrechts-Universität zu Kiel, Kiel, Germany; ^4^Institute for Clinical Molecular Biology, Christian-Albrechts-Universität zu Kiel, Kiel, Germany; ^5^Research Group Proteomics, Institute for Experimental Medicine, Christian-Albrechts-Universität zu Kiel, Kiel, Germany; ^6^Max Planck Institute for Evolutionary Biology, Plön, Germany

**Keywords:** *Caenorhabditis elegans*, microbiota, *Ochrobactrum*, transcriptomics, RNA-Seq, metabolic network model

## Abstract

The biology of all organisms is influenced by the associated community of microorganisms. In spite of its importance, it is usually not well understood how exactly this microbiota affects host functions and what are the underlying molecular processes. To rectify this knowledge gap, we took advantage of the nematode *Caenorhabditis elegans* as a tractable, experimental model system and assessed the inducible transcriptome response after colonization with members of its native microbiota. For this study, we focused on two isolates of the genus *Ochrobactrum*. These bacteria are known to be abundant in the nematode’s microbiota and are capable of colonizing and persisting in the nematode gut, even under stressful conditions. The transcriptome response was assessed across development and three time points of adult life, using general and *C. elegans*-specific enrichment analyses to identify affected functions. Our assessment revealed an influence of the microbiota members on the nematode’s dietary response, development, fertility, immunity, and energy metabolism. This response is mainly regulated by a GATA transcription factor, most likely ELT-2, as indicated by the enrichment of (i) the GATA motif in the promoter regions of inducible genes and (ii) of ELT-2 targets among the differentially expressed genes. We compared our transcriptome results with a corresponding previously characterized proteome data set, highlighting a significant overlap in the differentially expressed genes, the affected functions, and ELT-2 target genes. Our analysis further identified a core set of 86 genes that consistently responded to the microbiota members across development and adult life, including several C-type lectin-like genes and genes known to be involved in energy metabolism or fertility. We additionally assessed the consequences of induced gene expression with the help of metabolic network model analysis, using a previously established metabolic network for *C. elegans*. This analysis complemented the enrichment analyses by revealing an influence of the *Ochrobactrum* isolates on *C. elegans* energy metabolism and furthermore metabolism of specific amino acids, fatty acids, and also folate biosynthesis. Our findings highlight the multifaceted impact of naturally colonizing microbiota isolates on *C. elegans* life history and thereby provide a framework for further analysis of microbiota-mediated host functions.

## Introduction

All multicellular organisms live in close association with microbial communities, the so-called microbiota (McFall-Ngai et al., 2013). The microbiota appears to influence various biological functions of the host, for example food digestion and metabolism (Nicholson et al., 2012), development (Sampson and Mazmanian, 2015), immune defense (Tilg and Moschen, 2015), and aging (Heintz and Mair, 2014). It is also often linked to disease, such as obesity (Shen et al., 2013), liver cirrhosis (Abu-Shanab and Quigley, 2010), and even cancer (Garrett, 2015). However, the microbiota’s involvement in determining host traits is often based on correlation and exact information on its causal effects is usually absent due to missing possibilities for experimental manipulation. Well-established animal models may allow for such manipulations. Previous work with the fruitfly *Drosophila melanogaster* has indeed used an experimental approach to dissect the microbiota’s influence on host functions. These studies revealed that the microbiota affects gut morphology and physiological functions through changes in epithelial renewal rate, cellular spacing, and the distribution of different cell types in the epithelium (Broderick et al., 2014), that host genotype interacts with the microbiota to determine *Drosophila* nutritional state, as inferred from lipid content (Chaston et al., 2016), and that specific microbiota members enhance development and increase fly fitness (Pais et al., 2018). Moreover, microbiota members were demonstrated to interact with each other to influence host behavior, specifically olfactory and egg laying behaviors, mediated mainly through the odorant receptor *Or42b* (Fischer et al., 2017).

Another widely used model organism is the nematode *Caenorhabditis elegans*. Its native microbiota includes a species-rich community of mainly Gammaproteobacteria and Bacteriodetes, including taxa from the genera *Enterobacter*, *Pseudomonas*, and *Ochrobactrum* (Berg et al., 2016a; Dirksen et al., 2016; Samuel et al., 2016; Zhang et al., 2017). Most of the associated bacteria can be cultivated and are hence available for experimentation. Some of the cultivable isolates were already used to demonstrate their influence on *C. elegans* fitness under stress conditions, for example changes in nematode population growth under high osmolarity or temperature stress (Dirksen et al., 2016). Moreover, several distinct bacteria such as isolates of the genera *Pseudomonas*, *Enterobacter*, and *Gluconobacter*, were identified to enhance *C. elegans*’ immune defense against pathogens (Montalvo-Katz et al., 2013; Berg et al., 2016b, 2019; Dirksen et al., 2016; Samuel et al., 2016; Kissoyan et al., 2019). To date, it is yet unclear how exactly the microbiota affects host molecular mechanisms to influence *C. elegans* life history characteristics.

The objectives of the current study were to fill this knowledge gap and obtain first insights into the effects of microbiota representatives on *C. elegans* molecular processes. For this study, we focused on two microbiota members of the genus *Ochrobactrum*, the isolates MYb71 and MYb237. These bacteria show the particular ability to colonize the nematode gut, even under adverse environmental conditions, and thereby form persistent associations with *C. elegans* (Dirksen et al., 2016). To assess the bacteria’s influence on the host, we performed a whole-genome transcriptome analysis across nematode development and adult life, using RNA sequencing. We used complementary types of enrichment analysis, including usage of a *C. elegans*-specific gene expression database (i.e., WormExp; Yang et al., 2015b) and an assessment of over-represented transcription factor binding sites (Shi et al., 2011), in order to characterize the affected biological functions and the signaling processes likely involved. We further compared our new data with a recently published proteome analysis of related material (Cassidy et al., 2018), in order to evaluate whether the inducible transcriptome and proteome are consistent with each other and point to similar underlying processes. We further used the expression data for a reconstruction of the inducible metabolic activities in the worm gut, using the recently established metabolic network for *C. elegans* (Gebauer et al., 2016).

## Materials and Methods

### *C. elegans*, Bacteria Strains, and Phenotypic Assays

The canonical *C. elegans* laboratory strain N2 was used for all assays and generally maintained following standard procedures (Stiernagle, 2006). N2 was originally obtained from the CGC (Caenorhabditis Genetics Center), which is funded by NIH (National Institutes of Health) Office of Research Infrastructure Programs (P40 OD010440). We focused on this particular strain for our first analysis of the inducible *C. elegans* response, because it is well characterized under laboratory conditions and almost universally used across *C. elegans* laboratories, thus allowing us to compare our results with the comprehensive literature on N2.

Three bacterial strains were used. The two Gram-negative bacteria *Ochrobactrum anthropi* strain MYb71 and *Ochrobactrum pituitosum* strain MYb237 are members of the native microbiota of *C. elegans*, obtained from the *C. elegans* isolate MY316 collected from a rotten apple in Kiel, Germany (Dirksen et al., 2016). The bacteria were freshly thawed for each experiment and cultured for 2 days at 25°C on tryptic soy agar (TSA). Fresh colonies were used to produce liquid cultures in tryptic soy broth (TSB) at 28°C in a shaking incubator for approximately 42 h. The *Escherichia coli* strain OP50 was used as a control and cultured in TSB at 37°C in a shaking incubator overnight.

The induction of gene expression *in vivo* for ELT-2 down-stream target genes *spp-3* and *spp-5* was determined using transcriptional reporter strains. The promoters of *spp-3* (intergenic region between *spp-3* and *spp-2*) and *spp-5* (∼1.7 kb upstream of open reading frame) were amplified from genomic DNA (primer sequences upon request) and fused to the GFP amplicon of the Fire vector pPD95.75 by fusion PCR [pPD95_75 was a gift from Andrew Fire; Addgene plasmid # 1494^1^; RRID:Addgene_1494; (Hobert, 2002)]. The fusion constructs were injected into the gonads of young adult worms at a concentration of 10 ng/μl into mutant strain HT1593, *unc-119(ed3)* from the CGC. As co-injection marker, the *unc-119* rescue plasmid pPK605 at a concentration of 20 ng/μl was employed (pPK605 was a gift from Patricia Kuwabara, Addgene plasmid # 38148^2^; RRID:Addgene_38148).

For comparing the gene expression in the transgenic strains worms were exposed to MYb71, MYb237, or OP50 from the L4 stage for 24 h. Randomly picked worms were immobilized by 25 mM tetramisole and grouped on agar slides. Pictures were taken with a fixed exposure time using Zeiss Axio Observer Z.1(Carl Zeiss AG, Jena, Germany) und assembled with ImageJ (Version 1.52g) package Fiji (Schindelin et al., 2012).

To analyze the involvement of *elt-2* on *C. elegans* fitness, we exposed an *elt-2* overexpression strain and its control (Mann et al., 2016) to MYb71, MYb237, or *E. coli* OP50. Assay plates without peptone (peptone-free medium, PFM) were inoculated with a bacterial suspension in PBS at OD_600_ 10. Individual L4 worms were placed onto the bacterial lawn, transferred every day to fresh plates until they stopped laying eggs. The hatched offspring per individual worm was scored daily and added up to the total number of hatched offspring per worm. Replicates with worms that died before the end of their egg laying period were excluded. Statistical analyses were performed using R Studio (Version 1.0.136) and the “multcomp” package (Hothorn et al., 2015).

### Transcriptomic Analysis of the Inducible *C. elegans* Response by RNA-Seq

N2 worms were maintained for a minimum of two generations on PFM plates inoculated with the bacteria used for the experiments in order to allow adjustment to the bacteria. The transcriptome of *C. elegans* N2 was analyzed for six time points to cover various life stages: 6 h (second larval stage, L2), 24 h (L3), 48 h (L4), 72 h (1-day old adults, Ad1), 120 h (3-day old adults, Ad3) and 216 h (7-day old adults, Ad7). The worms were grown on 9 cm PFM plates with a 700 μl bacterial lawn (OD_600_ 10) of either MYb71, MYb237, or *E. coli* OP50. The worm stage was synchronized by bleaching. For each replicate 500 to 2000 synchronized hermaphrodites at the first larval stage (L1) were pipetted onto the bacterial lawn. The worms were maintained at 20°C and harvested after the indicated periods. To separate the initially added worms from their offspring and to ensure sufficient food, worms were transferred to new plates every 2 days starting from first day of adulthood. Worm stages which did not produce eggs (L2, L3, and L4) were washed from the plates with M9 buffer and centrifuged to obtain a worm pellet. The supernatant was removed and 700 μl TRIzol^TM^ (Thermo Fisher Scientific, Waltham, MA, United States) was added. Adult worms of Ad1, Ad3, and Ad7 were picked directly into 700 μl TRIzol^TM^ to separate the initially placed worms from their offspring. All worms in TRIzol^TM^ were five times frozen in liquid nitrogen and thawed at 46°C in a thermo shaker to break open the cuticle. Subsequently, the samples were frozen and stored at −80°C until the total RNA was extracted using the NucleoSpin RNA Kit (Macherey-Nagel, Düren, Germany). The transcriptome was analyzed for three replicates from independent runs of the exposure experiment. The only exception refers to L2 nematodes exposed to MYb237, for which only two independent replicates had sufficient amounts of RNA. All assays were performed without current knowledge of strain identity by using codes for labeling of plates and tubes that do not directly relate to the treatment and thereby leaves the experimenter unbiased during performance of the laboratory work. In addition, all treatment combinations were evaluated in parallel and in randomized order to further avoid any observer bias. RNA libraries were prepared for sequencing using standard Illumina protocols (TruSeq RNA v2; catalog number RS-122-2001). Libraries were sequenced on an Illumina HiSeq 2000 sequencing machine with paired-end strategy at read length of 100 nucleotides (HiSeq 3000/4000 PE and SBS Cluster Kits; catalog numbers PE-410-1001, FC-410-1003). The raw data is available from the GEO database (Edgar et al., 2001; Barrett et al., 2012) under the GSE number GSE111364.

After removal of adaptor sequences and low quality reads via Trimmomatic (Bolger et al., 2014), RNA-Seq reads were mapped to the *C. elegans* genome (Wormbase version WS235^3^) by STAR 2.5.3a (Dobin et al., 2013) under default settings. Transcript abundance (read counts per gene) was extracted via HTSeq (Anders et al., 2015). Differential expression analysis was performed by aFold from ABSSeq (Yang et al., 2016b). The log2 transformed fold-changes (*Ochrobactrum* vs. *E. coli* OP50) were taken as input for K-means cluster analysis using cluster 3.0 (de Hoon et al., 2004). We assessed variation in gene expression with the help of K-means cluster analysis, because this approach allows us to identify distinct groups of genes with similar expression patterns (D’haeseleer, 2005; Uygun et al., 2016) that are likely regulated through the same transcription factor(s) or other regulatory element(s). The identified distinct gene sets are then best suited for subsequent down-stream analyses, such as analysis of enriched transcription factor binding sites or enriched functions (see below). We compared different numbers of K-means clusters using the Akaike information criterion (Kodinariya and Makwana, 2013), in order to identify the most appropriate cluster number for our data (i.e., the one with lowest AIC value; Supplementary Figure S1). A heat map was generated by TreeView version 1.1.4r3 (Saldanha, 2004). Core *Ochrobactrum* responsive genes were detected via aFold using a linear model with the factors bacterial treatment and developmental time, in order to account for variation due to colonization with the different bacteria and also due to different developmental and adult life stages.

### Gene Ontology, Gene Set, and Motif Enrichment Analysis

Gene ontology (GO) analysis was performed using DAVID with a cut-off of FDR < 0.05 (Huang et al., 2009). A taxon-specific gene set enrichment analysis was performed using WormExp (Yang et al., 2015b), a web-based analysis tool for *C. elegans*, containing all of the available gene expression data sets for this nematode, thus allowing characterization of species-specific expression patterns. Only gene sets with FDR < 0.05 were considered to be significant. Motif analysis was carried out on the promoter regions, −600 bp and 250 bp relative to transcription start sites (TSS), of genes in each group. *De novo* motif discovery was performed using AMD (Shi et al., 2011).

### Transcriptome-Proteome Comparison

We compared our transcriptome results with the corresponding, previously published proteome data employing an isobaric labeling/LC-MS approach (Cassidy et al., 2018). The proteome data was generated from exposure experiments that were performed in almost identical form than those used for the transcriptome analysis. The main differences were that for the proteomics approach worms were grown on 15 cm plates (instead of 9 cm plates), Merck filters were used to separate larvae from the focal nematodes (instead of individual transfer of the focal worms with the help of worm-pickers), and only a single time-point was included, namely the young adult stage (i.e., after 72 h exposure of worms to the bacteria).

### Metabolic Network Analysis

Context-specific metabolic networks based on transcriptomic and proteomic data were reconstructed as described previously (Gebauer et al., 2016). Briefly, we used a two-step procedure in which first gene expression states were binarized into *on* and *off* and subsequently these states were used to derive activity of metabolic pathways through mapping to a genome-scale reconstruction of *C. elegans* metabolism using the iMAT procedure (Zur et al., 2010). In the first step, the procedure uses false discovery rate-adjusted *p*-values and fold-changes from differential expression analyses of the transcriptomic data to derive for each time point and condition the most likely activity state of a gene. While in the original procedure (Gebauer et al., 2016) this only involved comparisons to adjacent time points, we extended the approach by also comparing, for each time point, gene expression between all conditions. Differential gene expression analysis was performed using DESeq2 with standard parameters (Love et al., 2014). Only genes for which at least one comparison yielded an adjusted *p*-value below 0.05 across all comparisons were considered for the second step. The expression state of all other genes was left open in the second step of the analysis. In the second step, the iMAT-procedure (Zur et al., 2010) is used to derive a context-specific metabolic network that obeys the constraints of the network (steady state, flux bounds), maximizes the utilization of reactions associated with genes determined as *on* in the first step and minimized the utilization of reactions associated with genes determined as *off*. To determine changes in the activity of metabolic pathways, we used the subsystem annotation of each reaction present in the network reconstruction and counted the number of active reactions associated with each pathway for each condition and time point.

We next identified key enzymes involved in the response to bacterial colonization, using the metabolic transformation algorithm [MTA, (Yizhak et al., 2013)]. The metabolic transformation algorithm is able to identify reaction knockouts that are best able to transform a metabolic network from a given source state to a desired target state. We modified MTA in two points. First, we used the context-specific metabolic networks we reconstructed as described above as source state instead of the purely iMAT-derived metabolic networks used in the original approach (Yizhak et al., 2013) to maximize comparability of the identified key enzymes to the context-specific metabolic networks we derived. Second, to facilitate interpretation, we did not consider single-reaction knockouts but rather gene knockouts. Thus, we did not assess the ability of knockouts of specific reactions to move the source network toward the target state but rather we performed knockouts on the level of genes. Thus, we tested for each metabolic gene present in the reconstruction whether its knockout blocked any reaction and performed MTA on the metabolic network after constraining the flux through all correspondingly blocked reactions to zero.

We performed two sets of MTA runs on the transcriptomic and the proteomic data: one for the transition between growth of either *Ochrobactrum* species to growth on *E. coli* OP50 and vice versa. For each set of runs we performed MTA runs for every time point and each comparison. Thus, for the analysis of the metabolic transition between growth on *Ochrobactrum* to growth on *E. coli* OP50, we considered for each time point either of the two *Ochrobactrum*-growth specific metabolic networks as source state and the *E. coli* OP50-specific metabolic network as target state. MTA returns for each gene a score indicating to which extent the knockout of this gene shifts the source state toward the target state. Moreover, MTA determines a threshold score at which the knockout of a gene is considered to lead to a significant shift toward the target state. Since the absolute values of MTA-scores change between runs (but rarely their order), we summarized each run by setting all runs with an MTA-score equal or below the cut-off threshold to zero, replacing the remaining scores with their rank in the ordered list and normalizing values to a maximum of 1 (highest MTA score) and a minimum of 0. For each comparison we performed five bootstrap runs in which we randomly removed 10% of gene expression values before performing MTA to assess robustness of results. MTA-scores were summarized through averaging the rank-normalized scores for each gene across all runs of a set and subsequent normalization of scores to a range from 0 to 1. Subsequently, an overall MTA score for each gene was determined by summing MTA-scores for the transition from growth on *Ochrobactrum* to growth on *E. coli* OP50 using transcriptomic and proteomic data and subtracting the scores for the opposite transition. As a result, we obtained an overall MTA score which is positive if a gene mediates the transition from growth on *Ochrobactrum* to growth on *E. coli* OP50 and negative in the other direction.

## Results and Discussion

### Transcriptional Variation Is Determined by Nematode Development and Microbial Exposure

**FIGURE 1:**
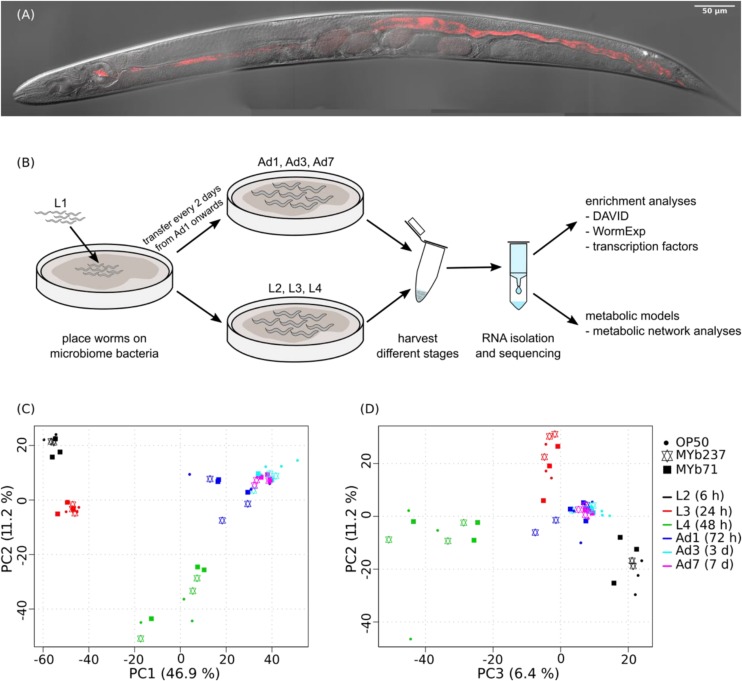
Colonized *C. elegans* individual, workflow and principal component analysis of transcriptomic variation. **(A)** Colonization of *C. elegans* strain N2 by *Ochrobactrum* MYb71, highlighting distribution of the microbiota isolate (labeled with red fluorescence) throughout the pharynx and gut lumen. See also Supplementary Movie S1. **(B)** Workflow. **(C,D)** Variation was assessed for N2 fed with *E. coli* OP50 (indicated by filled cycle) or colonized by *Ochrobactrum* isolates MYb237 (triangles up and down) or MYb71 (filled squares) at six time points including the second larval stage (L2, 6 h), L3 (24 h), L4 (48 h), 1-day old adults (Ad1), Ad3 (3 days), and Ad7 (7 days), as indicated by different colors.

The *C. elegans* microbiota isolates *Ochrobactrum* MYb71 and *Ochrobactrum* MYb237 are able to efficiently colonize the nematode gut [Figure 1A and Supplementary Movie S1, and also Dirksen et al. (2016)]. To assess the effects of MYb71 and MYb237 on molecular processes in the *C. elegans* host, we performed transcriptome analysis using RNAseq across three developmental stages (L2, L3, and L4) and three time points during adulthood (day 1, day 3, and day 7). The standard laboratory food bacterium *E. coli* OP50 served as control (Figure 1B). We first used principal component analysis (PCA) to explore transcriptomic variation across treatments and time points. We found that the first three principal components separate different *C. elegans* developmental stages (Figures 1C,D), suggesting development as the main determinant of the overall variation in gene expression. Thus, colonization by the *Ochrobactrum* strains and the *E. coli* control have a smaller effect on overall gene expression variation.

**FIGURE 2:**
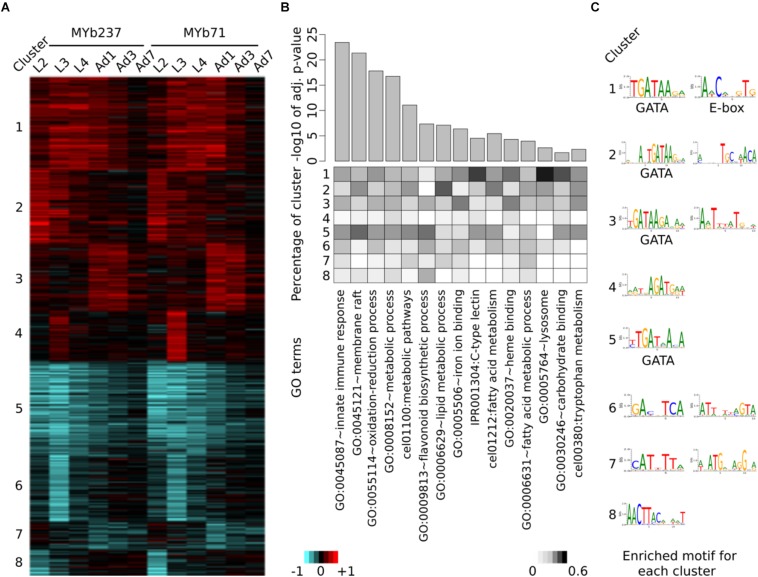
Induced differential gene expression in *C. elegans* after colonization with *Ochrobactrum* isolates. Differential expression is identified via comparison of worms colonized by the *Ochrobactrum* isolates *versus* those fed with *E. coli* OP50. **(A)** Co-regulation of differentially expressed genes. Eight clusters (indicated by the numbers on the left) of co-regulated genes were identified via K-means clustering. Red and blue colors refer to up and down regulation, respectively. Heatmap scale bars indicate fold changes at log_2_ scale, showing averages for the replicates of a particular treatment combination. **(B)** Enriched gene ontology (GO) terms of differentially expressed genes. GO enrichment analysis was performed by DAVID. 15 selected GO terms are shown with significance (top panel, bar plot with adjusted *p*-values). The percentage of genes, which contributed from each cluster to the identified GO terms is shown as a heatmap in the middle panel. **(C)** Enriched transcription factor binding sites (Motif) for each cluster. Motifs were detected by AMD at the promoter region of genes.

Next, we specifically assessed transcriptional variation by either *Ochrobactrum* strains versus *E. coli* for each time point, in order to explore the gene functions affected by these microbiota members. A total of 893 genes were significantly differentially expressed. Eight clusters were identified to best capture the variation present in this gene set, as inferred from a comparison of different cluster numbers with the Akaike information criterion (Supplementary Figure S1). The observed differential gene expression across these eight clusters (Figure 2A) highlight that the transcriptional response is indeed influenced by *Ochrobactrum* across time, whereby the two *Ochrobactrum* strains do not appear to vary much in inducible expression patterns (Figure 2A). This result may suggest that the two isolates are highly similar in their colonization characteristics and general interaction with *C. elegans*. Interestingly, they show these similarities, yet are clearly distinct at genome level. The publicly available genomes (NCBI Bioproject PRJNA400855) show 97% similarity in full 16S ribosomal RNA sequences (extracted with RNAmmer; (Lagesen et al., 2007), 85% average similarity in DNA sequences across the entire genomes [inferred with dnadiff from MUMmer ver. 4 (Kurtz et al., 2004)], and an overlap of 74% genes, as identified via PROKKA annotations (Seemann, 2014). For the transcriptome response of *C. elegans*, the clusters 1 and 5 refer to genes with strong differential expression across all time points (up- and down-regulated genes, respectively), while other clusters show differential expression at specific time points only. These distinct groups of *Ochrobactrum*-influenced differentially expressed genes possibly indicates that *Ochrobactrum* affects *C. elegans*’ life history in more than one way.

To uncover the affected biological processes for these possible different effects, we applied gene ontology (GO) enrichment analysis on each of the eight clusters separately. A variety of GO terms were found to be significantly over-represented among these genes (Supplementary Table S1). Several of the most significant GO terms are related to immunity, for example GO:0045087 (e.g., innate immune response) and IPR001304 for the C-type lectins (Pees et al., 2015), the latter term particularly over-represented among the up-regulated gene clusters (Figure 2B). These results are consistent with repeated previous reports of a link between the microbiota and the host immune system (Cullender et al., 2013; Tilg and Moschen, 2015; Thaiss et al., 2016). Moreover, many metabolism-related processes are significantly enriched (e.g., GO:0055114∼oxidation-reduction process and GO:0006629∼lipid metabolic process; Figure 2B and Supplementary Table S1). This may suggest an influence of *Ochrobactrum* on *C. elegans* metabolism, consistent with previous work on the alternative food bacterium *Comamonas* DA1877 (MacNeil et al., 2013). Interestingly, clusters 1 and 5 show a stronger relationship to the indicated GO terms than clusters related to only one time point. This may imply that the major effects of these microbiota members on *C. elegans* persist across the various life stages.

The complementary enrichment analysis with WormExp (Yang et al., 2015b) revealed significant over-representation of gene sets, which were related to pathogen infection [e.g., nematocidal *Bacillus thuringiensis* strain BT247 (Yang et al., 2015a), *Staphylococcus aureus* (Bond et al., 2014), *Pseudomonas aeruginosa* strain UCBPP-PA14 (Nakad et al., 2016); Table 1 and Supplementary Table S1]. Moreover, data sets related to the GATA transcription factor gene *elt-2* [RNAi (Schieber and Chandel, 2014), ChIP-Seq targets (Mann et al., 2016)] or the E-box transcription factor gene *hlh-30* [study on *S. aureus* (Visvikis et al., 2014) and *hlh-30* mutant (Grove et al., 2009)] were also enriched, suggesting an involvement of these transcription factors in the nematode’s response to the microbiota. Additional over-represented gene sets relate to *C. elegans* dietary responses, for example gene sets related to the insulin-like pathway [*daf-2* (Knutson et al., 2016) and *daf-16* (McElwee et al., 2004)], fasting (Lee et al., 2014, 1), and starvation (Mueller et al., 2014). We further found an enrichment of the worm’s response to the previously studied food bacterium *Comamonas* DA1877 (MacNeil et al., 2013). Overall, our results suggest that the *Ochrobactrum* isolates affect dietary responses, metabolic processes, and also interact with the immune system.

**TABLE 1:** Enriched WormExp gene sets for differentially expressed genes upon *Ochrobactrum* colonization.

**Category**	**Gene set**	**Counts**	**Bonferroni**
Microbes	down by *B. thuringiensis* at 6 h (BT247, 1:10) (Yang)	232	4.13E-149
TF Targets	low-complexity *elt-2* targets	338	4.68E-133
DAF/Insulin/food	down by *daf-2* mutant (Knutson)	171	7.24E-130
Mutants	down by *elt-2* RNAi under normoxia	141	4.78E-90
Microbes	up by PA14, 12 h	106	1.29E-81
DAF/Insulin/food	down by fasting (Lee)	90	8.03E-65
Microbes	down by Bt toxin, Cry5B	110	7.36E-64
Microbes	down by PA14, 12 h	63	7.59E-62
Microbes	down by *S. aureus* (Bond)	84	2.05E-53
Microbes	down by *B. thuringiensis* (BT247), 12 h	157	2.32E-49
DAF/Insulin/food	down by starved (Mueller)	170	4.55E-48
Microbes	down fed by *E. coli* HT115 vs. OP50	38	3.25E-47
DAF/Insulin/food	up by *daf-16* mutant (McElwee)	90	1.84E-46
Microbes	up by *B. thuringiensis* at 6 h (BT247, 1:10) (Yang)	112	2.35E-41
DAF/Insulin/food	down by *daf-16* mutant (McElwee)	95	3.11E-39
Microbes	up by *S. aureus*, dependent on *hlh-30* (Visvikis)	100	1.07E-38
Microbes	up by *S. aureus* (Bond)	85	5.18E-36
DAF/Insulin/food	down fed by *Comamonas* DA1877 vs. OP50 young adult	67	8.49E-33
DAF/Insulin/food	up by fasting (Lee)	52	1.39E-25
DAF/Insulin/food	up by starved (Mueller)	140	3.55E-25
DAF/Insulin/food	up fed by *Comamonas* DA1877 vs. OP50 young adult	31	1.06E-23
Mutants	up by *elt-2* RNAi under normoxia	39	3.64E-18
Microbes	up fed by *E. coli* HT115 vs. OP50	19	1.21E-16
DAF/Insulin/food	up by *daf-2* mutant (Knutson)	41	2.90E-13
Microbes	up by Bt toxin, Cry5B	46	4.86E-12
Mutants	down *hlh-30* mutant	27	7.65E-12

*The enriched gene sets (second column) are ordered according to their significance, as indicated by the Bonferroni-adjusted *p*-values (right column). The column counts shows the number of genes present in the current data set and that from the database. Detailed results are provided in Supplementary Table S1.*

The presence of co-regulated gene clusters, as inferred through our cluster analysis, could be caused by transcription factors. To explore this idea, we performed *de novo* motif enrichment analysis on promoter regions of the genes within each cluster. We identified one or two informative transcription factor binding motifs for each cluster (Figure 2C). However, only two of them have been previously characterized for *C. elegans*: the GATA motif with consensus sequence GATAA and the E-box with the sequence motif CACGTG. GATA transcription factors are known to play a role in immunity (Shapira et al., 2006), intestine development [mainly through ELT-2 (McGhee et al., 2007)], and aging [mainly through ELT-3, ELT-5, and ELT-6 (Budovskaya et al., 2008)]. The GATA motif is enriched in clusters 1, 2, 3, and 5, which generally showed an over-representation of GO terms and gene sets related to above characteristics, especially immunity (Figure 2B and Table 1). E-box transcription factors were shown to shape *C. elegans* immunity [e.g., *hlh-30* (Visvikis et al., 2014)] and muscle development (Grove et al., 2009). The corresponding motif was only identified for cluster 1, which similarly produced an enrichment for immunity-related GO terms. As the motif analysis is corroborated by the enriched gene expression sets for mutants of known GATA and E-box transcription factors, we conclude that these regulators play a central role in coordinating the response of *C. elegans* to its microbiota members.

### Signature Genes in the *C. elegans* Response to *Ochrobactru*m

**FIGURE 3:**
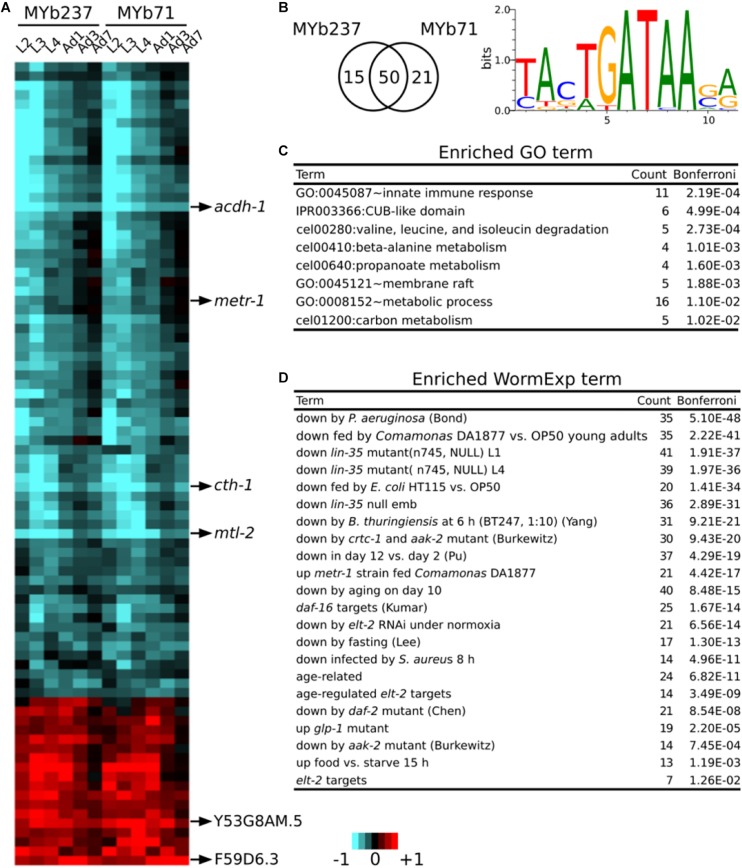
Core responsive genes. **(A)** Differential gene expression of 86 core responsive genes are shown using a heatmap. Genes with the strongest change of expression are marked on the right. Red and blue colors refer to up and down regulation, respectively. Heatmap scale bars indicate fold changes at log_2_ scale, always showing averages for the replicates of a particular treatment. **(B)** Venn diagram showing the overlap of core responsive gens induced upon colonization by MYb71 and MYb237 and the enriched binding motif. **(C)** Enriched GO terms. GO enrichment analysis was performed by DAVID. **(D)** Enriched WormExp gene sets.

To identify genes specific for the response to the microbiota members only, we employed a model-based statistical analysis (based on the linear model-option in aFold; Yang et al., 2016b), in which we statistically accounted for the factors bacterial treatment and time. Based on this approach, we extracted the genes, which specifically respond to the presence of *Ochrobactrum* (Figure 3A), irrespective of any major variation in gene expression across worm development. Please note that some of the identified genes still show some variation across time, indicating that the time effect cannot be fully removed through the model-based analysis. This analysis revealed a total of 86 differentially regulated genes: 65 differentially expressed in the presence of MYb237 and 71 in the presence of MYb71 (Figure 3B). 50 of these genes are differentially expressed in the presence of both *Ochrobactrum* isolates, while 15 genes respond specifically to MYb237 and 21 to MYb71 (Figure 3B). This overlap of more than 70% of the genes confirms our above notion that these two *Ochrobactrum* isolates induce a similar expression response. Of the total of 86 genes, 18 and 59 were consistently up- and down-regulated, respectively (Figure 3A). Of these genes, six showed a more than twofold change in expression. They may therefore be suited as indicator markers for the *C. elegans* response toward *Ochrobactrum* colonization. They include the four down-regulated genes *acdh-1*, *metr-1*, *cth-1*, and *mtl-2*, and the two up-regulated genes Y53G8AM.5 and F59D6.3. *acdh-1* and *metr-1* were previously identified as reporters for a dietary response (MacNeil et al., 2013; Watson et al., 2013). The down-regulated gene *mtl-2* is known to play a role in regulating growth and fertility (Freedman et al., 1993). *cth-1* encodes a putative cystathionine gamma-lyase, which may contribute to metabolic processes.

A motif enrichment analysis on these 86 genes again suggests that the microbiota response is controlled by a GATA transcription factor (Figure 3B). This is additionally supported by the significantly over-represented gene set that is downstream of the GATA transcription factor gene *elt-2* (Figure 3D and Supplementary Table S2). Our enrichment analysis generally yielded results that are consistent with above analysis (cf. Figure 2B). They indicate a potential interaction between *Ochrobactrum* and immunity and also the involvement of the dietary response, the microbe’s influence on metabolic pathways, energy production, and the response to the bacterial food source *Comamonas* DA1877 (Figures 3C,D and Supplementary Table S2). Moreover, in this analysis, we also found enriched gene sets related to development [i.e., downstream targets of *lin-35* (Kirienko and Fay, 2007)] and fertility [i.e., downstream targets of *glp-1* (Gracida and Eckmann, 2013)]. The latter suggest that the microbiota additionally affects these two characteristics in *C. elegans*.

### Concordance Between the *C. elegans* Proteome and Transcriptome Responses to *Ochrobactru*m

**FIGURE 4:**
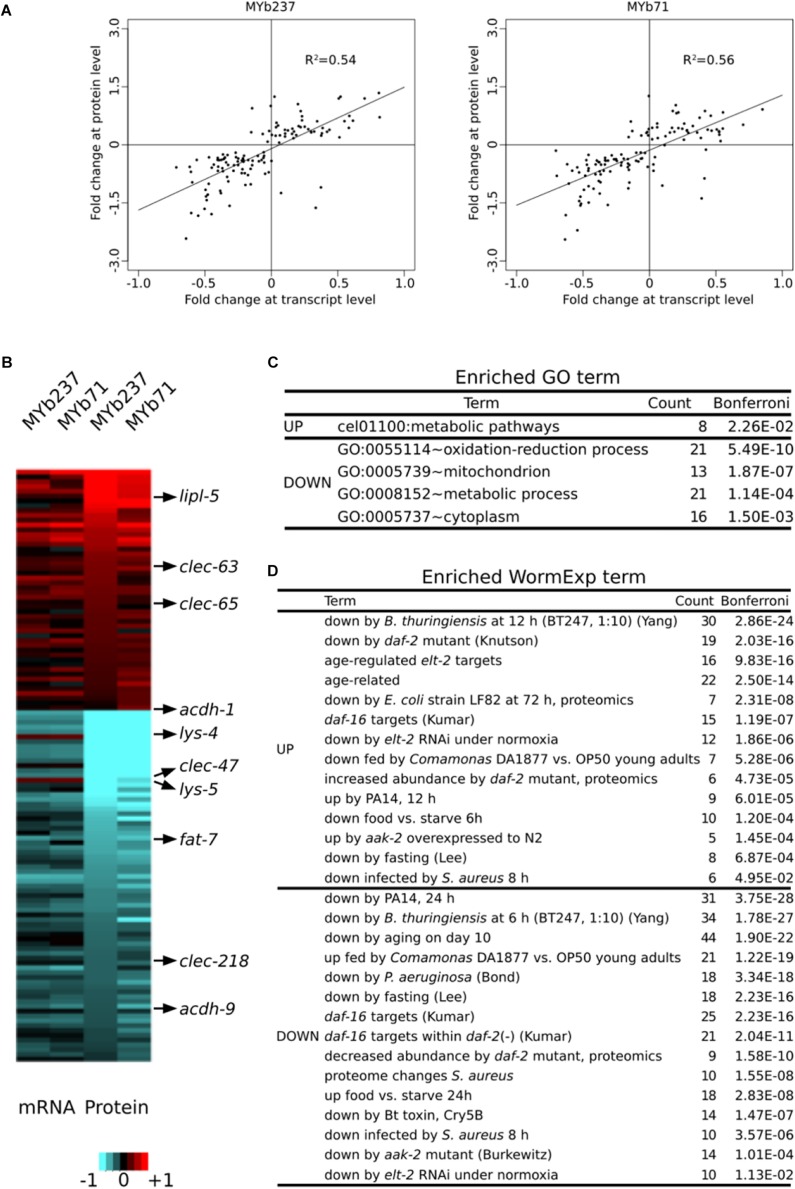
Comparison of differential gene expression at transcript and protein levels. **(A)** Comparison of expression levels at transcript and protein level. Data are only shown for genes with significant differential expression at protein level. Results are shown separately upon exposure to either MYb71 or MYb237. **(B)** Expression changes of genes at transcript and protein levels at the 1-day old adult stage (Ad1, 72 h). Red and blue colors refer to up and down regulation, respectively, or higher and lower protein abundances. Heatmap scale bars indicate fold changes at log_2_ scale, showing averages across replicates per treatment. A selection of genes with functional information is shown on the right. **(C)** Enriched GO terms. GO enrichment analysis was performed by DAVID. **(D)** Enriched WormExp gene sets.

We next compared the transcriptome response at the first adult time point to a corresponding proteome data set, which was obtained for the same time point upon colonization with *Ochrobactrum* (Cassidy et al., 2018). This proteome data set revealed significant differential abundance of 123 out of more than 3,600 quantified proteins. Of these, 50 had higher and 73 had lower abundance. These differentially abundant proteins showed consistent changes at the transcript level upon colonization of nematodes to both MYb71 and MYb237 and the correlation between proteome and corresponding transcriptome was highly significant (Pearson rank correlation, *R*^2^ ≥ 0.54, *p* < 0.0001; Figures 4A,B). In detail, 40 genes showed consistently higher (80% of the 50 up-regulated proteins) and 67 consistently lower abundance (91.8% of the 73 down-regulated proteins) in both the transcriptome and proteome data sets. One example is the short-chain acyl-CoA dehydrogenase gene, *acdh-1*, which was consistently and strongly down-regulated in the presence of the two *Ochrobactrum* isolates. Similarly, four C-type lectin-like genes produced consistent and significant expression changes at transcript and protein levels (up-regulation: *clec-63* and *clec-65*, down-regulation: *clec-47* and *clec-218*). These results were in line with the enriched GO term of C-type lectin genes (Figure 2B), suggesting an important role of C-type lectins in mediating the nematode’s interaction with specific microbiota members, which are highly numerous in nematodes and could contribute to recognition of microbe-associated molecular patterns (MAMPs) (Pees et al., 2015).

An enrichment analysis of the 123 differentially expressed proteins revealed several significantly enriched functions. These included GO terms related to metabolism and energy production, such as the terms cel01100:Metabolic pathways, GO:0008152∼metabolic process, and GO:0005739∼mitochondrion (Figure 4C and Supplementary Table S3). The enriched term GO:0055114∼oxidation-reduction process could similarly indicate a role in energy metabolism or, alternatively, stress response. The WormExp analysis yielded similar results as above, including significant over-representation of gene sets related to immunity, dietary response, aging, and that controlled by *elt-2*. We also found enrichment of genes controlled by *aak-2*, which is part of the AMPK pathway (Burkewitz et al., 2015; Hou et al., 2016) and thus further supports the possible influence of the microbiota members on *C. elegans* energy production (Figure 4D). Taken together, the independently generated proteome data set corroborates several findings of the transcriptome data analysis, highlighting the likely importance of the indicated underlying processes.

### The GATA Transcription Factor ELT-2 Appears to Influence the Response of *C. elegans* to *Ochrobactru*m

**FIGURE 5:**
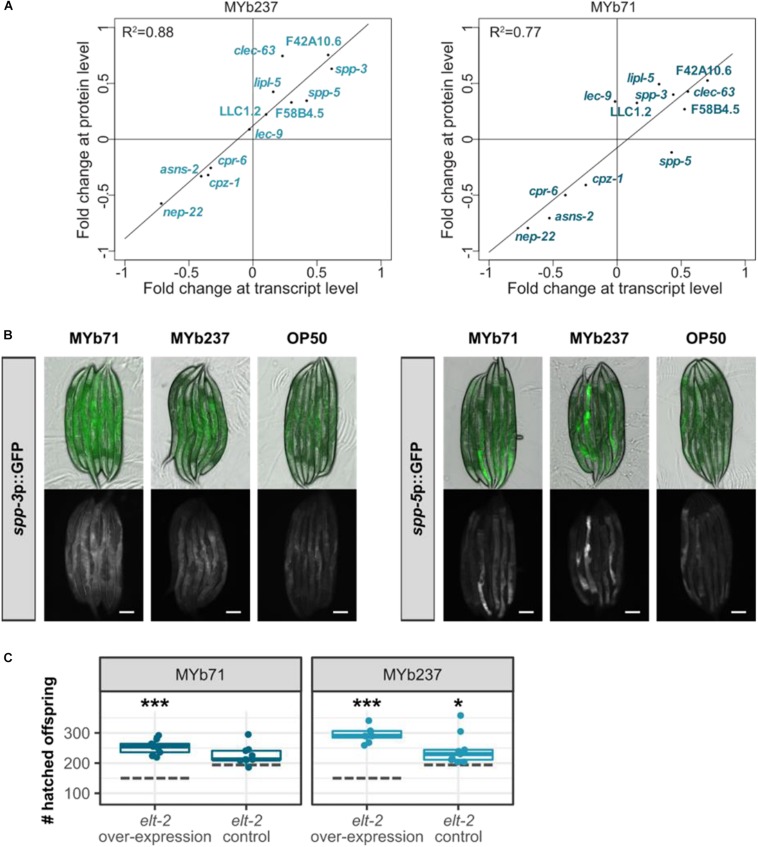
Influence of GATA transcription factor ELT-2 on transcriptomic response of *C. elegans* to *Ochrobactrum*. **(A)** Comparison of fold change at transcript and protein level. Data are only shown for a subset of genes, which are known to be ELT-2 downstream targets (Block and Shapira, 2015; Yang et al., 2015b; Wiesenfahrt et al., 2016). Results are shown separately upon colonization with either MYb71 or MYb237. **(B)** *In vivo* expression of ELT-2 target genes *spp-3* and *spp-5* on MYb71 and MYb237 compared to *E. coli* OP50. Pictures were taken with 1-day old adults at same exposure time. The scale bar represents 100 μm. **(C)** Fitness of an *elt-2* overexpression strain and its control (Mann et al., 2016) determined as number of hatched offspring per individual. Dashed gray lines represent the median of the respective strain on *E. coli* OP50. ^∗∗∗^*p* < 0.001, ^*^p < 0.05, Dunnett’s test, *n* = 4–9.

To further validate the involvement of GATA transcription factor ELT-2 mediating the response to *Ochrobactrum*, as consistently indicated by our enrichment analyses, we chose exemplarily a subset of known ELT-2 down-stream target genes (Block and Shapira, 2015; Wiesenfahrt et al., 2016) using WormExp (Yang et al., 2015b) and focused on those, which were differentially regulated in the proteome data set. The fold changes of these ELT-2 target genes were significantly correlated at both the protein as well as transcriptome level upon colonization with the two *Ochrobactrum* strains (Pearson correlation, *R*^2^ = 0.88, *p* < 0.0001 for the response to MYb237; *R*^2^ = 0.77, *p* = 0.0001 for that to MYb71; Figure 5A). Two of these ELT-2 target genes, antimicrobial peptide encoding genes *spp-3* and *spp-5* (Roeder et al., 2010; Hoeckendorf and Leippe, 2012), were additionally assessed in their *in vivo* expression using transcriptional reporters. We found that the expression of both genes can be induced in the intestine upon colonization with the two *Ochrobactrum* strains (Figure 5B), which confirms their up-regulation found in our transcriptomic data. Moreover, we tested the influence of ELT-2 on worm fitness as measured in hatched offspring per worm upon *Ochrobactrum* exposure using an *elt-2* overexpression strain. Our results show that the overexpression of *elt-2* affects bacteria-mediated worm fitness (Figure 5C).

Taken together, our results uniformly indicate an influence of ELT-2 on the nematode’s response to *Ochrobactrum*, and are thus consistent with the findings of our enrichment analyses. ELT-2 is known to control gene expression in the intestine (McGhee et al., 2007), where the microbiota resides, thus providing ample opportunity for direct interactions between bacterial molecules and upstream regulators of ELT-2 and, conversely, between ELT-2 downstream factors and the colonizing bacteria. Moreover, ELT-2 was suggested to act as central regulator of inducible gene expression against pathogenic bacteria in the adult intestine (Block and Shapira, 2015; Yang et al., 2016a), thereby providing a link to the enriched set of immune-related genes. However, *Ochrobactrum* does not behave as a pathogen, as it neither enhances nematode mortality (compared to the standard food source *E. coli*) nor causes any obvious damage during intestinal colonization (Figure 1A). Therefore, the enrichment of immune-related genes may indicate multiple functions of the pathogen-responsive genes, which could be involved in both digestion and pathogen degradation or the recognition of any gut-colonizing microbe [e.g., possible for the enriched group of C-type lectins; (Pees et al., 2015)]. This enriched category may also reflect the previous notion that immune-related genes are generally involved in coordinating composition of the host-associated microbes (McFall-Ngai, 2007; Eberl, 2010).

### Metabolic Network Analysis Indicates Colonization-Specific Changes in Fatty Acid, Amino Acid, Folate, and Energy Metabolism

Since we observed considerable metabolism-associated changes in response to bacterial colonization, we performed a more detailed analysis of metabolic changes associated to bacterial colonization. To this end, we derived context-specific metabolic networks by mapping the transcriptomic and proteomic data to a genome-scale reconstruction of *C. elegans* metabolism. This method comprises two steps that initially discretizes gene expression states into on and off based on differential expression between conditions. In the second step, a subnetwork of the metabolic network is determined that can carry flux and maximizes the utilization of reactions catalyzed by enzymes that are active based on the first step while minimizing the utilization of reactions catalyzed by enzymes that are inactive (Gebauer et al., 2016).

**FIGURE 6:**
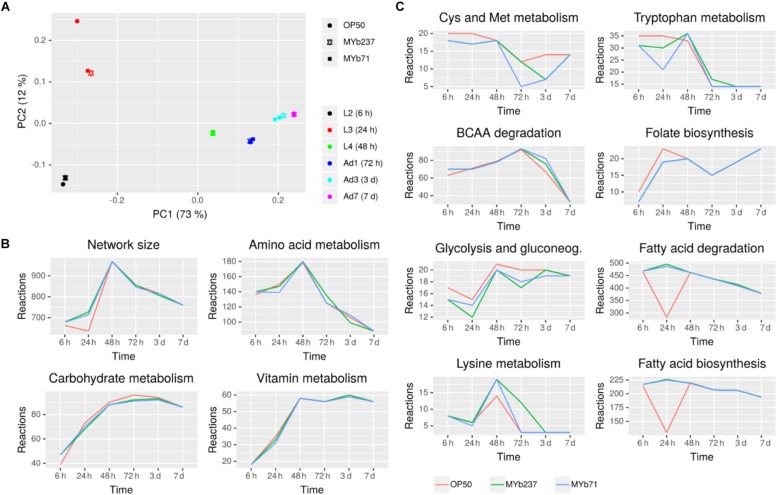
Metabolic response of *C. elegans* to bacterial colonization and exposure. **(A)** Principal component analysis of context-specific metabolic networks. **(B)** Changes in the number of active reactions in top-level pathways across developmental stages and bacterial treatment. **(C)** Changes in the number of active reactions in individual metabolic pathways. Only eight pathways with highest absolute difference in the number of active reactions between *Ochrobactrum* (MYb237 and MYb71) and *E. coli* OP50 exposure across all time points are shown. Numbers of active reactions in all subsystems are provided in Supplementary Tables S4, S5.

In a first step, we compared the reconstructed context-specific networks of the different developmental stages upon exposure to the different bacteria separately (Figure 6A). In agreement with the expression-based analysis, we found that developmental stage had the strongest impact on metabolic activity. Bacterial colonization affected metabolism mostly during larval development and had only little impact in adult worms. On a global scale, amino acid metabolism, carbohydrate metabolism, and vitamin metabolism were most strongly affected by bacterial colonization (Figure 6B and Supplementary Tables S4, S5). Differences among bacterial effects were found for fatty acid metabolism, the metabolism of various amino acids (branched-chain amino acids, cysteine/methionine metabolism, tryptophan metabolism, lysine metabolism), and also folate (Figure 6C). Intriguingly, folate metabolism has previously been observed to be a key process involved in the modulation of host physiology and lifespan by food microbes (Cabreiro et al., 2013). Moreover, branched-chain amino acids, cysteine as well as methionine and tryptophan are important modulators of *C. elegans* nutritional and stress responses (van der Goot et al., 2012; Lee et al., 2015; Mansfeld et al., 2015; Gebauer et al., 2016).

In a second step, we used the metabolic transformation algorithm (Yizhak et al., 2013) to identify enzymes that most strongly contributed to the metabolic shifts associated with bacterial colonization (Supplementary Table S6). The metabolic transformation algorithm identifies knockouts in a metabolic network that shift the metabolic network from a specific source state to a desired target state. Thus, this algorithm allows us to assess which enzymatic processes mediate the metabolic transition from *C. elegans* growth on the *Ochrobactrum* strains toward growth on *E. coli* OP50 and *vice versa*. In order to ensure robustness of the results, we used combined data sets of the transcriptomic and proteomic results for these analyses. For the transition from *E. coli* to *Ochrobactrum* (i.e., requirements for growth on the microbiota bacteria), we observed a considerable number of potential key mediators in N-glycan biosynthesis, serotonin/octopamine biosynthesis and co-factor metabolism. For N-glycan biosynthesis, several genes in N-glycan precursor biosynthesis including *algn-1* – *algn-3*, *algn-5*, *algn-7* and *algn-10* – *algn-13* were identified. N-glycans have been implicated in the interaction with microbial pathogens in *C. elegans* (Shi et al., 2006; Butschi et al., 2010) and commensals in higher organisms (Koropatkin et al., 2012). Thus, our results may indicate a role of N-glycans also in the interaction of *C. elegans* with its microbiota members. For serotonin/octopamine metabolism, we identified tyrosine decarboxylase (*tdc-1*) as well as tyramine beta-hydroxylase (*tbh-1*) involved in octopamine biosynthesis and tryptophan hydroxylase (*tph-1*) involved in serotonin biosynthesis as key mediators of metabolic transitions. Serotonin and octopamine are antagonists that are important modulators of *C. elegans* behavior and energy metabolism (Niacaris and Avery, 2003; Suo et al., 2009; Noble et al., 2013). For the transition from *Ochrobactrum* to *E. coli* OP50 (i.e., requirement for growth on *E. coli*), we identified only two enzymes as key mediators: a predicted adenosine kinase, R07H5.8, and C05C10.3 involved in ketone body metabolism as well as the first step in mevalonate/terpenoid metabolism. Interestingly, adenosine kinase catalyzes the conversion of adenosine and ATP to AMP and ADP and thereby influences central aspects of *C. elegans* physiology via AMPK-signaling. These results provide a novel framework for further exploration of these metabolic influences and may thus stimulate new research on the physiology and metabolism of *C. elegans*.

## Conclusion

We here present the first study on the transcriptional response of *C. elegans* toward colonizing members of its native microbiota, both from the genus *Ochrobactrum*. We found that the response to *Ochrobactrum* involves several biological processes, including immunity, aging, fertility, and development, and the three connected characteristics dietary response, metabolism, and energy production. We identified an *Ochrobactrum*-exclusive signature of 86 differentially expressed genes, some of which are directly related to above functions and could serve as indicator genes for the worm’s response to this microbiota taxon. We additionally identified a significant overlap between the *Ochrobactrum*-mediated response at transcript and protein levels, thereby providing one of the few examples in *C. elegans* which could directly connect these two levels within one study set-up. Our results consistently suggest that the GATA transcription factor ELT-2 is a key regulator of the nematode’s response to *Ochrobactrum*. Moreover, metabolic network analysis confirms the influence of *Ochrobactrum* on energy metabolism, and furthermore metabolism of specific amino acids, fatty acids, and also folate biosynthesis.

Taken together, our work provides a first global assessment of the response of *C. elegans* to colonization with members of its native microbiota. While focused on the description of this response and investigation of ELT-2 as one key regulator, our results yield a reference framework for future studies on the function of the nematode’s interaction with its native microbiota.

## Ethics Statement

This study used the invertebrate animal *Caenorhabditis elegans*, which does not fall under any legal restrictions.

## Author Contributions

WY, CP, BP, AT, ML, KD, CK, and HS designed the study. CP, BP, and PD performed the nematode experiments. CP isolated the RNA. PR coordinated the RNA sequencing. YW performed the transcriptome and proteome analysis. JZ, SW, and CK reconstructed and analyzed the metabolic network models. All authors discussed and interpreted the data. WY, BP, CP, CK, and HS wrote the manuscript.

## Conflict of Interest Statement

The authors declare that the research was conducted in the absence of any commercial or financial relationships that could be construed as a potential conflict of interest.

## Footnotes

1 http://n2t.net/addgene:1494

2 http://n2t.net/addgene:38148

3 www.wormbase.org

## Supplementary Material

FIGURE S1 Identification of the most appropriate number of clusters for K-means cluster analysis. Comparison of different K-means cluster numbers using the Akaike information criterion. The analysis was performed with the significantly differentially expressed genes of the main transcriptome data set.

TABLE S1 Fold-change in expression of differentially expressed genes, gene ontology (GO) term enrichment, and WormExp enrichment.

TABLE S2 Core responsive genes induced upon *Ochrobactrum* colonization, GO term enrichment, and WormExp enrichment.

TABLE S3 Comparison of differential expression at transcript and protein levels and WormExp enrichment.

TABLE S4 Inferred active reactions in metabolic subsystems across developmental stages and different bacterial microbiota species.

TABLE S5 Inferred active reactions in individual metabolic pathways across developmental stages and different bacterial microbiota species.

TABLE S6 Identification of key enzymes mediating the response to *Ochrobactrum* species and *Escherichia coli.*

MOVIE S1 3D reconstruction of *Ochrobactrum* MYb71 colonization in a *C. elegans* N2 host via fluorescence *in situ* hybridization. Signal of general bacterial probe EUB338 is shown in red, nuclei stained with DAPI in blue. The individual shown is the same as in Figure 1A.
