# Supplementary figures and images for: The Inducible Response of the Nematode Caenorhabditis elegans to Members of Its Natural Microbiota Across Development and Adult Life
Source: Front Microbiol. 2019 Aug 7;10:1793. doi: 10.3389/fmicb.2019.01793 (PMC6693516; doi:10.3389/fmicb.2019.01793)

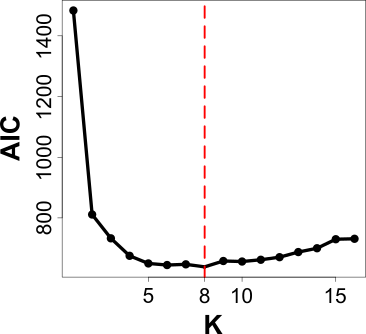

Supplement: FIGURE S1 — Identification of the most appropriate number of clusters for K-means cluster analysis. Comparison of different K-means cluster numbers using the Akaike information criterion. The analysis was performed with the significantly differentially expressed genes of the main transcriptome data set. [file Image_1.PNG]
